# Proteomics analysis of serum protein profiling in pancreatic cancer patients by DIGE: up-regulation of mannose-binding lectin 2 and myosin light chain kinase 2

**DOI:** 10.1186/1471-230X-10-68

**Published:** 2010-06-29

**Authors:** Yefei Rong, Dayong Jin, Chenrui Hou, Jianwen Hu, Wenchuan Wu, Xiaolin Ni, Dansong Wang, Wenhui Lou

**Affiliations:** 1 Pancreatic Cancer Group, Department of General Surgery, Zhongshan Hospital, Fudan University, Shanghai, China, 200032; 2 Research Center for Proteome Analysis, Key Laboratory of Proteomics, Institute of Biochemistry and Cell Biology, Shanghai Institutes for Biological Sciences, Chinese Academy of Sciences, Shanghai, China, 200032

## Abstract

**Background:**

Pancreatic cancer has significant morbidity and mortality worldwide. Good prognosis relies on an early diagnosis. The purpose of this study was to develop techniques for identifying cancer biomarkers in the serum of patients with pancreatic cancer.

**Methods:**

Serum samples from five individuals with pancreatic cancer and five individuals without cancer were compared. Highly abundant serum proteins were depleted by immuno-affinity column. Differential protein analysis was performed using 2-dimensional differential in-gel electrophoresis (2D-DIGE).

**Results:**

Among these protein spots, we found that 16 protein spots were differently expressed between the two mixtures; 8 of these were up-regulated and 8 were down-regulated in cancer. Mass spectrometry and database searching allowed the identification of the proteins corresponding to the gel spots. Up-regulation of mannose-binding lectin 2 and myosin light chain kinase 2, which have not previously been implicated in pancreatic cancer, were observed. In an independent series of serum samples from 16 patients with pancreatic cancer and 16 non-cancer-bearing controls, increased levels of mannose-binding lectin 2 and myosin light chain kinase 2 were confirmed by western blot.

**Conclusions:**

These results suggest that affinity column enrichment and DIGE can be used to identify proteins differentially expressed in serum from pancreatic cancer patients. These two proteins 'mannose-binding lectin 2 and myosin light chain kinase 2' might be potential biomarkers for the diagnosis of the pancreatic cancer.

## Background

Pancreatic ductal adenocarcinoma is the most common malignancy of the pancreas that arises from the ductal epithelium of the pancreas. Despite many efforts, conventional treatment approaches, including surgery, radiation, chemotherapy, or combinations of these, have had little impact on the outcome of this lethal disease. The overall prognosis for the disease is dismal: after resection, the median survival does not exceed 2 years with a 5-year survival rate less than 20% [1-3]. One major reason for this poor outcome is the late diagnosis of the disease and only about 10-20% of patients are eligible for resection [4,5]. The overall 5-year survival rate of patients with curative resection of pancreatic cancer is 25%. This can be improved to 40% when the tumor is operated in its early stages, for example, when the tumor size is less than 2 cm and there are no lymph node metastases [6,7]. Therefore, early diagnosis of pancreatic cancer is of paramount importance in clinical practice.

Serum is a preferred specimen for the early diagnosis of malignant tumors, because samples are available readily by less invasive methods. Although extensive efforts have been focused on finding candidates for serum tumor markers and many candidates have been listed, none of them has yet been proven to be clinically useful. These tumor markers are neither tumor-specific nor pancreatic cancer-specific. CA19-9 is the most widely used tumor marker in pancreatic cancer, however the specificity is low. It may also be elevated in benign conditions including pancreatitis, hepatitis and cirrhosis, as well as in gastric cancer and colon cancer [8-10]. Other markers such as CEA, CA125 are being studied, but not specifically for the diagnosis of pancreatic cancer.

Recent advances in proteomic technologies, including 2-dimentional differential in-gel electrophoresis (2D-DIGE) and improved MS, have provided new opportunities for identifying biomarkers and therapeutic targets for cancer treatment. DIGE is effective in separating complex protein samples and in quantifying protein levels between samples. Proteins of interest can then be identified by tandem mass spectrometry (MS/MS). By loading an internal common standard sample labeled with a different fluorescent dye, gel-to-gel variations are cancelled out and quantitative proteomic profiling can be achieved across multiple samples [11].

A major problem is that the most abundant serum proteins like albumin and immunoglobin comprise over eighty-five percent of total serum protein. Proteins of interest are probably present at significantly lower concentrations. The most abundant serum proteins make it difficult to run two-dimensional gels reproducibly. These abundant proteins also limit the amount of serum that can be loaded onto 2-D gels for analysis, and mask differential expression of lower-abundance proteins with similar molecular weights and isoelectric points [12,13]. In this study, we used an immuno-affinity column that effectively removed the highest abundance proteins from the serum of patients with pancreatic cancer and non-cancer-bearing control individuals. Samples were then analyzed by DIGE and MS/MS. These techniques had allowed for the identification and characterization of a number of potential protein biomarkers. Differential expression of the proteins of interest had been confirmed using western blot.

## Methods

### Patients and Serum sample preparation

Blood samples were obtained from five pancreatic cancer patients and five non-cancer-bearing individuals, and samples from an additional 16 pancreatic cancer and 16 non-cancer-bearing donors were collected for the further validation at Zhongshan Hospital (Fudan University, Shanghai, China). Informed consent was obtained from all donors and the protocol was approved by the institutional review board of the Zhongshan Hospital. Blood samples (5 ml) were obtained from the donors in tubes without additive and allowed to clot at room temperature for 40 min. Serum was separated by centrifugation at 3000 rpm for 10 min and stored at -80°C until use.

Serum samples were processed using the Agilent Multiple Affinity Removal Column (Multiple Affinity Removal Column, 4.6 mm × 50 mm; Agilent Technologies, Palo Alto, CA), that selectively removes albumin, IgG, IgA, antitrypsin, transferrin and haptoglobin from the serum sample. Samples were processed according to the manufacturer's instructions. For each sample, a low abundance fraction was collected and buffer-exchanged into 10 mM Tris-HCl pH 7.4 using 5000 Da molecular weight cutoff spin concentrators (Agilent Technologies, Palo Alto, CA). Protein quantification was performed using the Coomassie protein assay reagent (Pierce Biotechnology, Rock-ford, absorbance at 595 nm), with a Bradford protein assay. The bovine serum albumin was used as standard protein. Approximately 90% of total serum protein was removed by this method.

### Fluorescence labeling

The rationale for using a pooled internal standard with DIGE to control for gel-to-gel variation has been described previously in detail [11]. A total of 50 μg of serum protein was labeled at a minimal level with one of three CyDye DIGE Fluors (GE Healthcare, Piscataway, NJ). Individual serum samples from 3 groups (pooled internal standard, cancer and control) were labeled with Cy2, Cy3 and Cy5 respectively. The three dyes were designed to ensure that proteins common to each sample had the same relative mobility regardless of the dye used to tag them. CyDyes were reconstituted in anhydrous DMF and combined with samples at a ratio of 400 pmol of CyDye to 50 μg of protein. Labeling was performed on ice and in the dark for 30 min. The reaction was then quenched by incubating with 1.5 μL of 10 mM lysine on ice and in the dark for 10 min.

### 2D Gel Electrophoresis and Imaging

Three labeled protein samples (standard, cancer, and control sample) were combined. 1 mg of unlabeled pooled standard sample was processed separately by 2 D gel electrophoresis for purposes of protein identification. Proteins were focused on 13 cm, 3-10 immobilized pH gradient (IPG) strips (GE Healthcare, Piscataway, NJ) using an IPGphor focusing apparatus (GE Healthcare, Piscataway, NJ). IPG strips were then equilibrated in equilibration buffer (50 mM Tris-HCl, 6 M urea, 30% glycerol, 2% SDS) supplemented with 1% dithiothreitol(DTT) to maintain the fully reduced state of proteins, followed by 2.5% iodoacetamide to prevent reoxidation of thiol groups during electrophoresis. Proteins were separated on 10%Tris-glycine gels (ProtoGel, National Diagnostics, Atlanta, GA) using an Ettan DALT II System (GE Healthcare, Piscataway, NJ). The gels were scanned with a 2920-2 D MasterImager (GE Healthcare BioSciences). Spot detection, quantification and image matching were performed with Decyder software (GE Healthcare Bio-Sciences). Triplicate gels were run for each sample to reduce gel-to-gel variations. The emission filters were Blue 488 nm (Cy2), Green 532 nm(Cy3), and Red 633 nm(Cy5). The 2-D gel containing 1 mg of unlabeled pooled standard sample was fixed in 30% methanol, 7.5% acetic acid, and then Coomassie stained (Colloidal Blue stain kit, Invitrogen, Carlsbad, CA), and scanned with the same imager, with an excitation wave length of 633 nm. We ran triplicate gels for each sample to reduce the gel-to-gel variations.

### DIGE Analysis

Relative protein quantification across pancreatic cancer and control samples was performed using DeCyder Differential In Gel Analysis and Biological Variance Analysis software (Version 4.0, GE Healthcare, Piscataway, NJ). The Cy2-labeled pooled internal standard on every gel allowed accurate relative quantitation of protein spot features across different gels. Student's *t*-test and one-way ANOVA were used to calculate significant differences in relative abundances of protein spot-features in cancer sera compared with control sera.

### In-gel Digestion

Protein spot-features that were significantly increased or decreased (*p *< 0.05) in all cancer samples compared to control samples were chosen for further analysis. In-gel digestion was performed for protein spots excised by an automated spot picker (SpotPicker; GE Healthcare BioSciences). The 2 mm diameter gel plugs were washed in Milli-Q water for 15 min, and then washed three times in 25 mM NH_4_HCO_3_, 50% CH_3_CN for 30 min while vortex-mixing. Gel plugs were then dehydrated in 100% CH_3_CN for 10 min while vortex-mixing. The supernatant was removed, and gel plugs were allowed to air-dry for 1 h.12 μg of 1.5 μM trypsin (sequence-grade trypsin, Promega, Madison, WI) suspended in 25 mM NH_4_HCO_3 _was added, and gel plugs were allowed to re-hydrate for 30 min on ice. Gel plugs were placed at 37°C and allowed to digest overnight. 96-well plates were then gently centrifuged, and the supernatant was taken for MALDI-TOF or LTQ MS analysis.

### MS/MS and Data Analysis

Differentially expressed protein spots were excised from the gels. Trypsin peptide solutions were mixed at a 1:1 ratio with 5 mg/mL R-cyano-4-hydroxy-cinnamic acid (CHCA) matrix in 0.3% TFA, and spotted on stainless steel MALDI sample plates (Applied Biosystems, Framingham, MA). Peptide mixtures were then analyzed using MALDI-TOF (4700 Proteomics Analyzer, Applied Biosystems, Framingham, MA) or LTQ MS (ThermoQuest, San Jose, CA, USA) coupled with a Surveyor HPLC system (ThermoQuest). In MALDI-TOF MS, protein identification was performed using Global Proteome Server Explorer software (Applied Biosystems, Framingham, MA) with the NCBI Reference Sequence (RefSeq Release 5) human protein database. Identification was assigned to a protein spot feature if the protein score was calculated to be greater than 50, correlating to a confidence interval of 99%. In LTQ MS, the full scan ranged from M/Z 400 to 2000. Protein identification using MS/MS raw data was performed with SEQUEST software (University of Washington, licensed to Thermo Finnigan) based on the database of Swiss-prot (release 54.7). A relative molecular mass of 57 (57D) was added to the average molecular mass of cysteines in MS/MS data searching. Protein identification results were filtered with the Xcorr(1+≥ 1.9, 2+≥ 2.2, 3+≥ 3.75 ) and DelCn(≥ 0.1). *(Experiments of 2 D gel electrophoresis and protein identification by MS were completed by the Shanghai Institutes for Biological Sciences, Research Center for Proteome Analysis)*.

### Western Blot Analysis

Serum samples from an additional 16 pancreatic cancer and 16 non-cancer-bearing donors were collected for the western blot analysis. These serum samples were non-immuno-depleted and protein concentrations were determined using Bradford reagent (Sigma) according to the manufacturer's instructions. Equal amounts of protein (30 μg) were mixed with loading buffer ( 62.5 mM Tris-HCl, pH6.8, 10% glycerol, 2% SDS, 2%β-mercaptoethanol and bromphenol blue), boiled for 5 min and subjected to SDS-PAGE. Proteins were transferred to polyvinylidene difluoride membranes (Millipore, Bedford, MA). The membranes were blocked with 5% fat-free dry milk in Tris-buffered saline containing 0.05% Tween 20(TBST) for 1 h at room temperature and incubated overnight with primary antibodies in TBST with 1% bovine serum albumin. After washing with TBST for three times, the membranes were further incubated for 1 h at room temperature with corresponding horseradish peroxidase-conjugated secondary antibody in appropriate dilution and then followed by washing twice with TBST and once with TBS. The immuno-reactive protein bands were visualized by ECL kit (Pierce). Anti-MBL2, anti-MLCK2 and anti-β-actin antibodies were purchased from Santa Cruz Biotechnology. The expression of β-actin was used as loading control.

### Statistical Analysis

Two-sided, Student's *t*-tests were used to analyze differences in protein levels between cancer and control sera. A *p *value of less than 0.05 was considered statistically significant (SPSS V11.0).

## Results

### Proteome differential expression in serum between pancreatic cancer patients and control

In this study, 2-DE was carried out with the serum samples taken from both the experimental and control groups. The gels were digitized and analyzed by DeCyder 4.0 software.

### Identification of differentially expressed proteins by MS/MS

**Figure 1:**
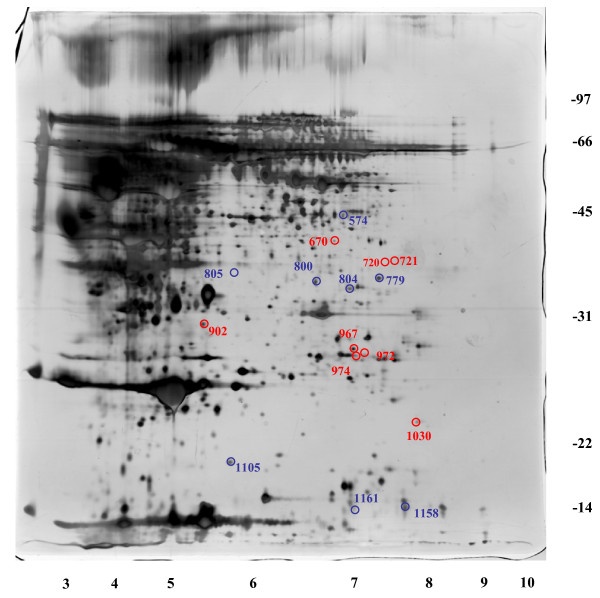
**Differentially expressed protein spots identified by DIGE analysis**. Eight protein spot-features were found to be significantly up-regulated (red) and eight were significantly down-regulated in the sera of patients with pancreatic cancer (blue).

In a pilot study, sera from 5 patients with pancreatic cancer were compared to the serum from 5 non-cancer-bearing donors (Table 1). Samples were processed by immuno-affinity depletion of the six most abundant serum proteins. Differential protein analysis was then performed using DIGE, as previously described. A total of 1254 and 1251 protein spots were detected in the experimental group and control group, respectively. Eight protein spot-features were found to be up-regulated significantly and eight were down-regulated significantly in the sera of patients with pancreatic cancer (Figure 1).

**Table 1:** Donor information of the sample set for DIGE

Case^a^	Age	Sex	Tumor location	Stage^b^
T1	51	M	Head	II
T2	50	M	Head	II
T3	62	F	Tail	III
T4	71	M	Head	II
T5	54	F	Tail	III
N1	40	F		
N2	44	M		
N3	55	M		
N4	65	F		
N5	60	M		

^a^T: pancreatic cancer patients; N: non-cancer bearing donors.

^b^The Union International Contre Le Cancer(UICC) classification.

All spot features of interest were trypsin-digested and submitted to MS/MS for identification. MALDI-TOF MS was used first for the identification of the protein spots. The results indicated that protein spots (1158, 805, 800, 779 and 574) had the protein mass fingerprints (PMF) and the corresponding proteins could be identified (Table 2). Protein spots (1105, 1161, 967, 902, 887, 804, 720 and 670) had protein mass finger prints, but did not correspond to proteins in the NCBI Reference Sequence human protein database. Then the LTQ-MS was used for the identification of these protein spots. Five proteins were identified as shown in the Table 3.

**Table 2:** Identification results of proteins differentially expressed in pancreatic cancer patients and cancer-free control (MALDI-TOF MS)

Spot	Protein name	**Accession no**.	MW	p*I*	Protein score	Sequence Coverage	Fold change (T/N)	*p*Value
574	amplified in osteosarcoma isoform 1 precursor	gi|5803109	75971	4.80	70	14%	-1.52	<0.05
779	unnamed protein product	gi|1335344	70723	5.53	78	14%	-2.05	<0.05
800	602153805F1 NIH_MGC_83 Homo sapiens cDNA clone	gi|11953201	32115	9.15	91	30%	-2.01	<0.05
805	AV723279 HTB Homo sapiens cDNA clone HTBAPBT7	gi|10826531	23047	9.77	98	24%	-1.94	<0.05
1158	serum amyloid A4	gi|10835095	14854	9.27	92	40%	-2.03	<0.05

**Table 3:** Identification results of proteins differentially expressed in pancreatic cancer patients and cancer-free control (LTQ-MS)

Spot	Protein name	Swiss-Prot ID	MW	p*I*	Sequence coverage (%)	Fold change(T/N)	*p*Value
720	MYLK2 Myosin light chain kinase 2, skeletal/cardiac muscle	Q9H1R3	64684.86	6.6	1.68	2.30	<0.05
902	MBL2 Mannose-binding protein C precursor	P11226	26143.49	5.39	8.87	1.57	<0.05
967	CA1 Carbonic anhydrase 1	P00915	28870.18	6.59	10.73	1.61	<0.05
1105	HPR Isoform 1 of Haptoglobin-related protein precursor	P00739	39007.42	6.41	3.45	-2.64	<0.05
1161	SAA4 Serum amyloid A-4 protein precursor	P35542	14806.71	9.27	8.46	-2.69	<0.05

### Elevated Levels of MBL2 and MLCK2 in pancreatic cancer serum

**Figure 2:**
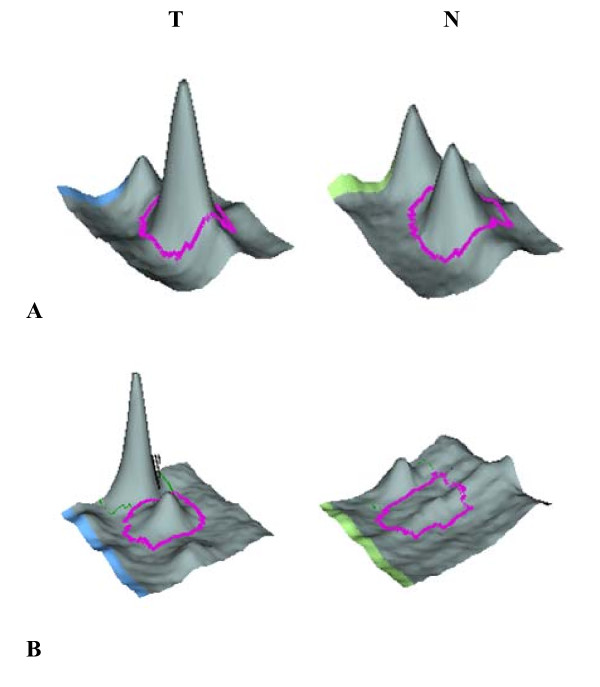
**3-D view of differentially expressed proteins**. 3-D view of differentially expressed proteins in the serum of pancreatic cancer patients and the cancer-free controls. The computational analysis of the images with the DyCyder software allowed for the detection of significant abundance changes based on the variance of the mean change within the cohort. MBL2 (A) and MLCK2 (B) were over-expressed in the pancreatic cancer patients' serum. T: the pancreatic patients; N: the controls.

**Figure 3:**
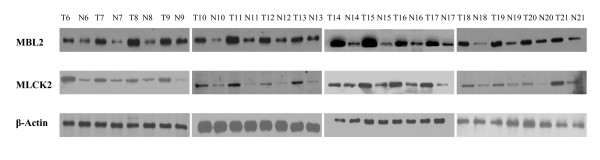
**Western blot analysis**. Western blot analysis confirms the DIGE findings of increased protein levels of MBL2 and MLCK2 in serum from pancreatic cancer patients (n = 16).

**Figure 4:**
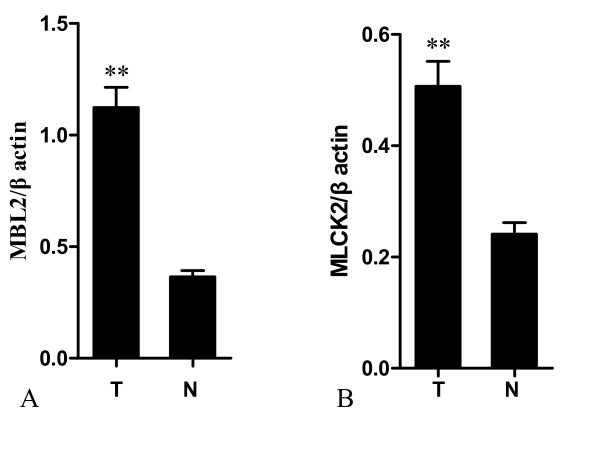
**The expression level of MBL2 and MLCK2**. The expression level of MBL2 and MLCK2 was assessed by analyzing the intensity of its bands normalized with β-actin. Comparison of serum protein levels between patients with pancreatic cancer and the cancer-free controls (n = 16) showed increased protein levels of MBL2 (A) and MLCK2 (B) (**p < 0.05).

Up-regulation of serum MBL2 and MLCK2 in pancreatic cancer was found by the DIGE and the 3 D spot images were shown in Figure 2. Western blot analysis was then performed for these two proteins in an independent set of serum samples. Serum from 16 pancreatic cancer patients and 16 non-caner-bearing controls were tested (Table 4). Western blots showed the consistent over-expressed of MBL2 and MLCK2 in individual pancreatic cancer patients compared with individual non-cancer-bearing donors (n = 16), and the Figure 3 showed the western blot results of 16 pairs of the pancreatic cancer sera and non-cancer-bearing controls' sera. Relative protein levels were then quantified using Imagequant software (GE healthcare, Piscataway, NJ), and statistical analysis was performed. Levels of these two proteins tested were significantly higher in the serum samples from cancer patients compared with controls (Figure 4, n = 16, *p *< 0.05).

**Table 4:** Donor information for the validation set

Case^a^	Age	Sex	Disease	Stage^b^
T6	58	F	Pancreatic adenocarcinoma	IV
T7	53	M	Pancreatic adenocarcinoma	III
T8	64	M	Pancreatic adenocarcinoma	II
T9	51	M	Pancreatic adenocarcinoma	III
T10	64	F	Pancreatic adenocarcinoma	III
T11	81	M	Pancreatic adenocarcinoma	III
T12	59	M	Pancreatic adenocarcinoma	II
T13	63	M	Pancreatic adenocarcinoma	III
T14	69	M	Pancreatic adenocarcinoma	III
T15	48	M	Pancreatic adenocarcinoma	III
T16	67	M	Pancreatic adenocarcinoma	IV
T17	56	M	Pancreatic adenocarcinoma	IV
T18	37	F	Pancreatic adenocarcinoma	III
T19	58	F	Pancreatic adenocarcinoma	III
T20	52	F	Pancreatic adenocarcinoma	IV
T21	62	F	Pancreatic adenocarcinoma	I
N6	45	F	Serous cystic pancreatic neoplasm	
N7	54	F	Somatostatin cell tumor	
N8	60	M	Islet cell tumor	
N9	54	M	Mucinous cystic pancreatic neoplasm	
N10	36	M	Pseudocyst	
N11	36	F	Serous cystic pancreatic neoplasm	
N12	70	M	Pseudocyst	
N13	53	M	Pancreatitis	
N14	59	F	Pancreatitis	
N15	50	F	Chronic pancreatitis	
N16	48	F	Intraductal papillary mucinous neoplasm	
N17	37	F	Normal	
N18	54	M	Normal	
N19	64	M	Normal	
N20	72	M	Normal	
N21	56	F	Normal	

^a ^T: pancreatic cancer patients; N: non-cancer bearing donors.

^b ^The Union International Contre Le Cancer(UICC) classification.

## Discussion

Currently, effective methods for the early detection of pancreatic cancer are still lacking. As a consequence, most patients are diagnosed in the late stage of the disease and the subsequent treatment is palliative rather than curative. At present, many serum markers for pancreatic cancer have been reported; including CEA, PNA-binding glycoproteins [14], hTert(telomerase catalytic subunit) [15] and matrix metalloproteinase-2(MMP-2)[16]. These markers lack adequate sensitivity and specificity and have limited utility as an indicator for the early, localized pancreatic disease. As early surgery is the only curative intervention, there is an urgent need to screen better serum biomarkers for the early detection of pancreatic cancer. Theoretically, it would be preferable to study patients exclusively in early stage disease; however, most pancreatic cancer patients are in the advanced stage when diagnosed. Therefore, some markers found in sera from patients with advanced stage pancreatic cancer may not be present in early stage cancer. It is reasonable to conclude, however, that some markers detected in advanced stage pancreatic cancer may also be present in early stage disease [13].

However, there was a large scale of non-interest proteins in the serum which made it difficult to analyze. In this study, we used the immuno-affinity column to remove the six most abundant proteins from serum samples. This technique proves to be both reproducible and effective in removing these highly abundant proteins as mentioned by Kenneth et al [13]. Immunoaffinity-depleted serum samples from patients with pancreatic cancer and controls were then analyzed using DIGE. Sixteen unique, differentially expressed proteins were identified by MALDI-TOF MS and LTQ MS. Further validation studies in an independent set of serum samples from 16 pancreatic cancer patients and 16 non-cancer-bearing controls demonstrated increased levels of MBL2 and MLCK2.

However not every protein that was elevated in the pilot study was validated in the independent set of serum samples; for example, carbonic anhydrase 1 which failed validation. It is not surprising that one or more of the candidate biomarkers identified in our pilot study would fail validation. This may have occurred for a few reasons. Only five cancer patients and non-cancer-bearing controls were analyzed in the pilot study. Differences among such a small number of samples quite easily could be unrelated to the disease state. One of the advantages of using DIGE, compared to other separation techniques such as multidimensional liquid chromatography, is that different levels of one isoform or one post-translationally modified form of a protein can be ascertained. These differences, however, may prove difficult to confirm using standard immunoblotting techniques, due to a lack of specific antibodies. As in our study, spot 779 was an unnamed protein and spot 800 and 805 could only be identified by the cDNA sequence in the protein database. Therefore it was difficult to validate these proteins and identifying the function of these proteins might need further study.

Proteomic analysis of serum protein profiles has allowed researchers to identify new serum markers of pancreatic cancer, but the identified proteins included common serum proteins and components of the acute phase response, which are not probably released from tumors. MBL2, which was found in this study to be increased in pancreatic cancer serum, has not been identified previously as a marker of pancreatic cancer. MBL2 is a calcium-dependent serum protein that plays a role in the innate immune response by binding to carbohydrates on the surface of a wide range of pathogens including viruses, bacteria, fungi, and protozoa where it can activate the complement system or act directly as an opsonin [17,18]. Recent studies found that polymorphisms in MBL2 have been associated with various tumors. Wang et al [19] reported that the codon 54 polymorphism of the MBL2 gene was associated with more advanced phenotypes of gastric cancer and the risk of gastric cancer in Japanese patients at 65 years of age or younger. Pine et al[20] reported that the functional Y/X polymorphism of MBL2 and MBL2 haplotypes and diplotypes appear to be associated with lung cancer survival among Caucasian patients. Caucasian lung cancer patients with low serum MBL levels were statistically significantly associated with improved survival time. In addition, the polymorphism of MBL2 might have influence on the risk of breast cancer in African-American women [21]. Another study, however, showed that the polymorphism of MBL2 had no relation with the hepatic cancer [22]. In our study, we showed for the first time that the serum levels of MBL2 were significantly higher in pancreatic cancer patients than in the non-cancer-bearing controls (Figure 4A), suggesting that MBL2 might represent a new serum marker for pancreatic cancer. However, whether it is associated with the development of pancreatic cancer need further study.

MLCK2, a calcium/calmodulin (cam) dependent enzyme, which expresses in adult skeletal muscle, was expressed at a significantly higher level in sera from pancreatic cancer than in the non-cancer-bearing control sera (Figure 4B). Thus, over-expression of this protein was tumor-specific and might be used for early diagnosis of pancreatic cancer. At present, there are few studies associated with the MLCK2. However, as the isoform of the MLCK2, MLCK has been studied extensively. MLCK is responsible for smooth muscle cell and non-muscle cell migration via phosphorylation of Ser19 and Thr18 on myosin light chains (MLC), which could facilitate myosin interaction with actin filaments[23,24]. Minamiya et al measured MLCK mRNA expression in tumor samples from 39 non-small cell lung cancer (NSCLC) patients. Their results showed that expression of MLCK was correlated with disease recurrence and distant metastasis in NSCLC [25]. Other researchers showed that inhibition of MLCK by its inhibitor ML-7 could effectively retard the proliferation and migration of the prostatic, breast and pancreatic cancer cells. This suggested the possibility that blocking of myosin II activity by a specific MLCK inhibitor may be a therapeutic strategy for preventing the invasion and metastasis of these tumors [26-28]. In accordance with these findings, our proteomic results suggested that MLCK2 might be a cancer-associated gene and a novel tumor marker of pancreatic cancer. Thus, the role of MLCK2 in carcinogenesis deserves further investigation.

## Conclusions

In the present study, we carried out DIGE proteomics analysis on sera from pancreatic cancer patients and its cancer-free controls. A total of 16 differentially expressed proteins were identified, of which 8 were over-regulated and 8 were under-regulated in the sera of pancreatic cancer patients. We further confirmed the expression levels of two up-regulated proteins (MBL2 and MLCK2) in the sera from pancreatic cancer patients using western blot analysis. MBL2 and MLCK2 were identified for the first time as serum biomarkers in pancreatic cancer. Serum MBL2 and MLCK2 measurement might be helpful in discriminating pancreatic adenocarcinoma from chronic pancreatitis and healthy controls. The newly identified proteins accompanied with the other serum biomarkers (CEA and CA19-9) might be useful for the early diagnosis of the pancreatic cancer.

## Competing interests

The authors declare that they have no competing interests.

## Authors' contributions

YFR gathered the serum sample and the clinical information and wrote the article. WCW and XLN helped in the gathering of the sera. DYJ, DSW and WHL performed the surgery. WHL designed this experiment and helped to edit the article. YFR, CRH and JWH performed the DIGE analysis, protein identification and the western blot validation. All authors read and approved the final manuscript.

## Pre-publication history

The pre-publication history for this paper can be accessed here:

http://www.biomedcentral.com/1471-230X/10/68/prepub
